# Genetic deletion of muscle RANK or selective inhibition of RANKL is not as effective as full-length OPG-fc in mitigating muscular dystrophy

**DOI:** 10.1186/s40478-018-0533-1

**Published:** 2018-04-24

**Authors:** Sébastien S. Dufresne, Antoine Boulanger-Piette, Sabrina Bossé, Anteneh Argaw, Dounia Hamoudi, Laetitia Marcadet, Daniel Gamu, Val A. Fajardo, Hideo Yagita, Josef M. Penninger, A. Russell Tupling, Jérôme Frenette

**Affiliations:** 1 Centre Hospitalier Universitaire de Québec–Centre de Recherche du Centre Hospitalier de l’Université Laval (CHUQ-CRCHUL), Université Laval, 2705 boulevard Laurier, RC-9500, Quebec City, QC G1V 4G2 Canada; 2 Department of Kinesiology, University of Waterloo, Waterloo, ON N2L 3G1 Canada; 3 Department of Immunology, Juntendo University, School of Medicine, Tokyo, Japan; 4 IMBA, Institute of Molecular Biotechnology of the Austrian Academy of Sciences, 1030 Vienna, Austria; 5 Département de Réadaptation, Faculté de Médecine, Université Laval, Quebec City, QC G1V 4G2 Canada

**Keywords:** Osteoprotegerin, Skeletal muscle, SERCA, Duchenne muscular dystrophy

## Abstract

Although there is a strong association between osteoporosis and skeletal muscle atrophy/dysfunction, the functional relevance of a particular biological pathway that regulates synchronously bone and skeletal muscle physiopathology is still elusive. Receptor-activator of nuclear factor κB (RANK), its ligand RANKL and the soluble decoy receptor osteoprotegerin (OPG) are the key regulators of osteoclast differentiation and bone remodelling. We thus hypothesized that RANK/RANKL/OPG, which is a key pathway for bone regulation, is involved in Duchenne muscular dystrophy (DMD) physiopathology. Our results show that muscle-specific RANK deletion (*mdx-RANK*^*mko*^) in dystrophin deficient *mdx* mice improves significantly specific force [54% gain in force] of EDL muscles with no protective effect against eccentric contraction-induced muscle dysfunction. In contrast, full-length OPG-Fc injections restore the force of dystrophic EDL muscles [162% gain in force], protect against eccentric contraction-induced muscle dysfunction ex vivo and significantly improve functional performance on downhill treadmill and post-exercise physical activity. Since OPG serves a soluble receptor for RANKL and as a decoy receptor for TRAIL, *mdx* mice were injected with anti-RANKL and anti-TRAIL antibodies to decipher the dual function of OPG. Injections of anti-RANKL and/or anti-TRAIL increase significantly the force of dystrophic EDL muscle [45% and 17% gains in force, respectively]. In agreement, truncated OPG-Fc that contains only RANKL domains produces similar gains, in terms of force production, than anti-RANKL treatments. To corroborate that full-length OPG-Fc also acts independently of RANK/RANKL pathway, dystrophin/RANK double-deficient mice were treated with full-length OPG-Fc for 10 days. Dystrophic EDL muscles exhibited a significant gain in force relative to untreated dystrophin/RANK double-deficient mice, indicating that the effect of full-length OPG-Fc is in part independent of the RANKL/RANK interaction. The sarco/endoplasmic reticulum Ca^2+^ ATPase (SERCA) activity is significantly depressed in dysfunctional and dystrophic muscles and full-length OPG-Fc treatment increased SERCA activity and SERCA-2a expression. These findings demonstrate the superiority of full-length OPG-Fc treatment relative to truncated OPG-Fc, anti-RANKL, anti-TRAIL or muscle RANK deletion in improving dystrophic muscle function, integrity and protection against eccentric contractions. In conclusion, full-length OPG-Fc represents an efficient alternative in the development of new treatments for muscular dystrophy in which a single therapeutic approach may be foreseeable to maintain both bone and skeletal muscle functions.

**Electronic supplementary material:**

The online version of this article (10.1186/s40478-018-0533-1) contains supplementary material, which is available to authorized users.

## Introduction

Bone and muscle have the ability to adjust their structures to meet their mechanical, hormonal, and metabolic environments. Osteoporosis and muscle atrophy/dysfunction occur simultaneously in a number of conditions, including spaceflight, extended bed rest, and several muscular and neuromuscular diseases. Local and systemic alterations in hormone and pro-inflammatory cytokine levels stimulate muscle and bone atrophy [25, 32]. Changes in intracellular Ca^2+^ concentrations regulate the physiological activities and expression of specific bone and muscle genes [15, 30]. Poor bone health and increased incidence of bone factures are well recognized clinically in Duchenne muscular dystrophy (DMD) patients suggesting cross-talks and mutual cooperative interactions between bones and dystrophic muscles [11]. However, the potential cellular and molecular mechanisms that may tie together bones and skeletal muscles during physiological and pathological conditions remain elusive.

The receptor-activator of nuclear factor κB ligand (RANKL), the membrane receptor RANK, and the soluble decoy receptor osteoprotegerin (OPG) are members of the tumor necrosis factor (TNF) superfamily that regulates bone remodelling [19, 27]. RANKL is expressed by osteoblasts, while RANK, its receptor, is expressed by pre-osteoclastic cells. The RANK/RANKL interaction induces the formation of multinucleated mature osteoclasts, ultimately causing bone resorption [21]. OPG, the third protagonist, is also produced by osteoblasts, binds to RANKL and exerts an inhibitory effect on the pre-osteoclastic differentiation process [2]. Structurally, the native OPG protein is highly conserved and contains four TNFR-like domains (RANKL binding sites), two death domains (tumour necrosis factor-related apoptosis-inducing ligand [TRAIL binding sites]), and a heparin-binding domain [31]. Thus, OPG serves as a decoy receptor for the RANKL and TRAIL and is a very efficient anti-resorptive and anti-apoptotic agent [3].

The focus of research in our laboratory is to decipher the potential cellular and molecular mechanisms that may tie together bones and skeletal muscles during physiological and pathological conditions. We first hypothesized that RANK/RANKL/OPG pathway, a key regulator of bone homeostasis and Ca^2+^ storage, would contribute in the regulation of skeletal muscle integrity and function during the course of muscular dystrophy. We previously demonstrated that daily full-length OPG-Fc treatment markedly improved muscle function and integrity in 5 week-old mdx mice [12]. The main objective of this study was to determine the specific contribution of muscle RANK, RANKL and TRAIL in muscular dystrophy. Using genetic and pharmacological approaches in young and adult dystrophic mice, we are able to show the unequivocal superior effects of full-length OPG-Fc in rescuing dystrophic muscles relative to selective muscle RANK deletion or anti-RANKL or anti-TRAIL treatments. Altogether, our results suggest that full-length OPG-Fc is a multifunctional protein that has the potential to impact on several different cellular processes with possibly profound implications for the treatment of DMD.

## Materials and methods

### Animals

Mice carrying the *RANK*^*floxed*^ or *RANK*^*del*^ alleles and muscle creatine kinase-cre (mck-cre) mice were backcrossed five times to a *C57BL/6* background before generating the *mck-cre RANK*^*del/floxed*^ (*RANK*^*mko*^) mice as previously described [13, 20]. Male wild-type (*C57BL/6*) and *mdx* dystrophic mice (*C57BL/10ScSn-Dmd*^*mdx*^*/J*) were purchased from the Jackson Laboratory (Bar Harbor, ME, USA) and bred at our animal facility. *RANK*^*mko*^ mice were also crossed with *mdx*-background mice to generate double deficient mice (dystrophin and RANK). Mice were screened for the desired genotype by PCR analysis. PCR products were amplified using primer pairs as listed in Additional file 1: Table S2. Dystrophic *mdx* mice were injected daily with full-length OPG-Fc [12] [i.p., 1 mg/kg/d R&D systems, MN, USA], PBS, anti-RANKL [39] [1 mg/kg/ every 3 d, clone IK22–5], anti-TRAIL [22] [1 mg/kg/every 3 d, clone H2B2] or truncated OPG-Fc [1 mg/kg/d, Syd Labs, MA, USA] from days 25 to 35 after birth. In another set of experiments, five six-month old *mdx* mice were injected daily, for 10 d, with full-length OPG-Fc [i.p. 1 mg/kg/d] followed by a downhill (eccentric) treadmill running protocol. *C57BL/6* mice were used as a control and injected daily with the same volume of phosphate-buffered saline (vehicle). At the end of the experimental procedures, mice were euthanized by cervical dislocation under anesthesia and skeletal muscles [*extensor digitorum longus* (EDL), *soleus* (Sol) and diaphragm (Dia)] were removed and stored at − 80 °C for future analysis. All procedures were approved by the Université Laval Research Center Animal Care and Use Committee, based on the Canadian Council on Animal Care guidelines. All data generated or analysed during this study are included in this published article and its Additional file 1.

### Immunofluorescence and staining

Transverse EDL muscle sections (10 μm) were cut (Leica Microsystems CM1850, Nussloch, Germany) in duplicate from the proximal and distal parts of the muscles. Tissue sections stained with hematoxylin and eosin (Sigma-Aldrich, St. Louis, MO, USA) were examined with an inverted microscope (Nikon, Ontario, Canada) and damage, regenerating and intact areas on approximately 100 myofibers per muscle were quantified with ImageJ software version 1.41 (National Institutes of Health, USA). The damaged area was defined as an area not occupied by normal or regenerating muscle fibers. Image series of EDL were taken using a confocal microscope (Axio Observer.Z1; Carl Zeiss, Germany) and acquired using a Quorum WaveFX spinning disc confocal system (Quorum Technologies, Ontario, Canada). Solid state laser lines 491 nm and 561 nm were used for excitation of green and red (Alexa-488 and Alexa-594), combined with appropriate BrightLine single-bandpass emission filters (536/40 nm and 624/40 nm, Semrock, NY, USA). z-series were acquired at the same time with DAPI fluorescence filter cube (Chroma Technology, VT, USA). The CCD camera used to capture the images was a Hamamatsu Image EM C-9100. Images were acquired and analyzed using Volocity software, version 4.2.1. Iterative restoration (deconvolution) was applied for the DAPI channel, using the same software.

### Western blots and qPCR

Skeletal muscles were homogenized in a lysis buffer containing 1 μg/ml, protease inhibitor cocktail (P8340; Sigma-Aldrich, Ontario, Canada), 20 mM Tris-base pH 7.5, 140 mM NaCl, 1 mM MgCl_2_, 1 mM CaCl_2_, 10% glycerol, 1% Igepal (Sigma-Aldrich, Ontario, Canada), 2 mM Na_3_VO_4_, 8.3 mM NaF and 0.2 mM PMSF. The protein content of the supernatant was measured using BCA protein assay kit (EMD chemical, Nussloch Germany). Protein homogenates were electrophoretically separated on SDS-polyacrylamide gels, transferred to polyvinylidene difluoride membranes (PVDF; Bio-Rad, CA, USA), blocked in 5% skim milk and incubated overnight at 4°C with the following primary antibodies (all from Santa Cruz Biotechnology): anti-SERCA-1a, anti-SERCA-2a anti-RANK and anti-GAPDH. The membranes were washed and incubated with appropriate HRP-conjugated secondary antibodies (Santa Cruz Biotechnology, CA, USA). Bands were revealed using the ECL-Plus chemiluminescent detection system (Perkin-Elmer, MA, USA). Images of the membranes were acquired, scanned, and analyzed using Quantity One software (v4.6.6, Bio-Rad). For RT-PCR analysis (Additional file 1: Table S2), skeletal muscles were rapidly put in RNAlater RNA Stabilization Reagent (Qiagen, MD, USA). Total RNA was isolated using the RNeasy Fibrous Tissue Mini Kit (Qiagen, MD, USA) according to manufacturer’s instructions. During isolation process, RNA samples were treated with RNase-Free DNase Set (Qiagen, MD, USA). RNA quality was assessed using an Agilent 2100 Bioanalyzer (Agilent Technologies, CA, USA) and quantified using a NanoDrop 1000 Spectrophotometer (NanoDrop Technologies, DE, USA). The expression of the RANK gene in each sample was compared to the housekeeping gene GAPDH. Measurements were performed in duplicate for each standard and muscle sample.

### Contractile properties

Mice were injected with buprenorphine (i.p. 0.1 mg/kg) and were anaesthetized with pentobarbital sodium (i.p. 50 mg/kg) 15 min later. The right Sol and EDL muscles were resected and attached to a 305B-LR dual-mode lever arm system controlled by dynamic muscle control unit and data acquisition software (Aurora Scientific, Aurora, ON, Canada) as described by Dufresne et al. (2016) [13]. For the eccentric contraction protocol, the muscles were set at optimal L0 and stimulated at 150 Hz for 700 ms. Five hundred ms into the stimulation protocol, the muscles were lengthened to 10% of L0 at 0.5 L0/s for 200 ms.

### Downhill running protocol

Six month-old *C57BL/6* and *mdx* mice were treated for 10 days with PBS (*n* = 12) or full-length OPG-Fc [1 mg/kg/d] (n = 12). From days 7 to 9, mice were trained for acclimatization on an horizontal (0% grade) motorized treadmill at 6, 8 and then 10 m/min for 5 min. Following the training protocol, mice ran on a downhill sloped (14-degree decline) at 10 m/min for 45 min. Mice were continually observed during the running protocol. Exhausted mice showing physical signs of discomfort were rested for 2 min. The running protocol was discontinued after 3 stops. Following the eccentric protocol, post-exercise mouse activity was measured by video tracking software in an open field for 24 h.

### SERCA activity

Frozen EDL, Sol and Dia muscles were homogenized with a ground-glass pestle in 5 volumes of 10 mM Tris/HCl (pH 8.3) supplemented with 0.3 M sucrose. SERCA activity was measured in A. R. Tupling’s laboratory with an assay adapted for a spectrophotometric plate reader according to Duhamel and colleagues (2007) [14] following the oxidation of NADH at 340 nm in assay buffer (pH 7.0) containing 1 mM EGTA, 10 mM phosphoenolpyruvate, 18 U/mL pyruvate kinase and lactate dehydrogenase, 0.2 mM NADH, 20 mM HEPES, 200 mM KCl, 15 mM MgCl_2_, 10 mM NaN_3_, and 5 mM ATP. The homogenized muscles and assay buffer were added to tubes containing 15 different concentrations of Ca^2+^ (between 7.6 and 4.7 pCa units) in the presence and absence of ionophore A23187 (4.2 μM). The absence of the ionophore causes back-inhibition of SERCA pumps. Then 0.3 mM NADH was added to start the reaction and the plate was read at a wavelength of 340 nm for 30 min at 37 °C. The different concentrations of Ca^2+^ were used to determine the maximal enzyme activity (Vmax) and pCa50. Lastly, cyclopiazonic acid (CPA; 40 μM), a highly specific SERCA inhibitor, was used to determine background activity.

### Statistical analyses

All values are expressed as means +/− SEM. The data were analyzed with Student’s t-test or one-way ANOVA with Tukey post hoc test (InStat). The levels of significance was set at *p < 0.05, **p < 0.01, and ***p < 0.001 for PBS-treated mdx.

## Results and discussion

### OPG-fc is superior to muscle-specific RANK deletion in mitigating muscular dystrophy

**Fig. 1:**
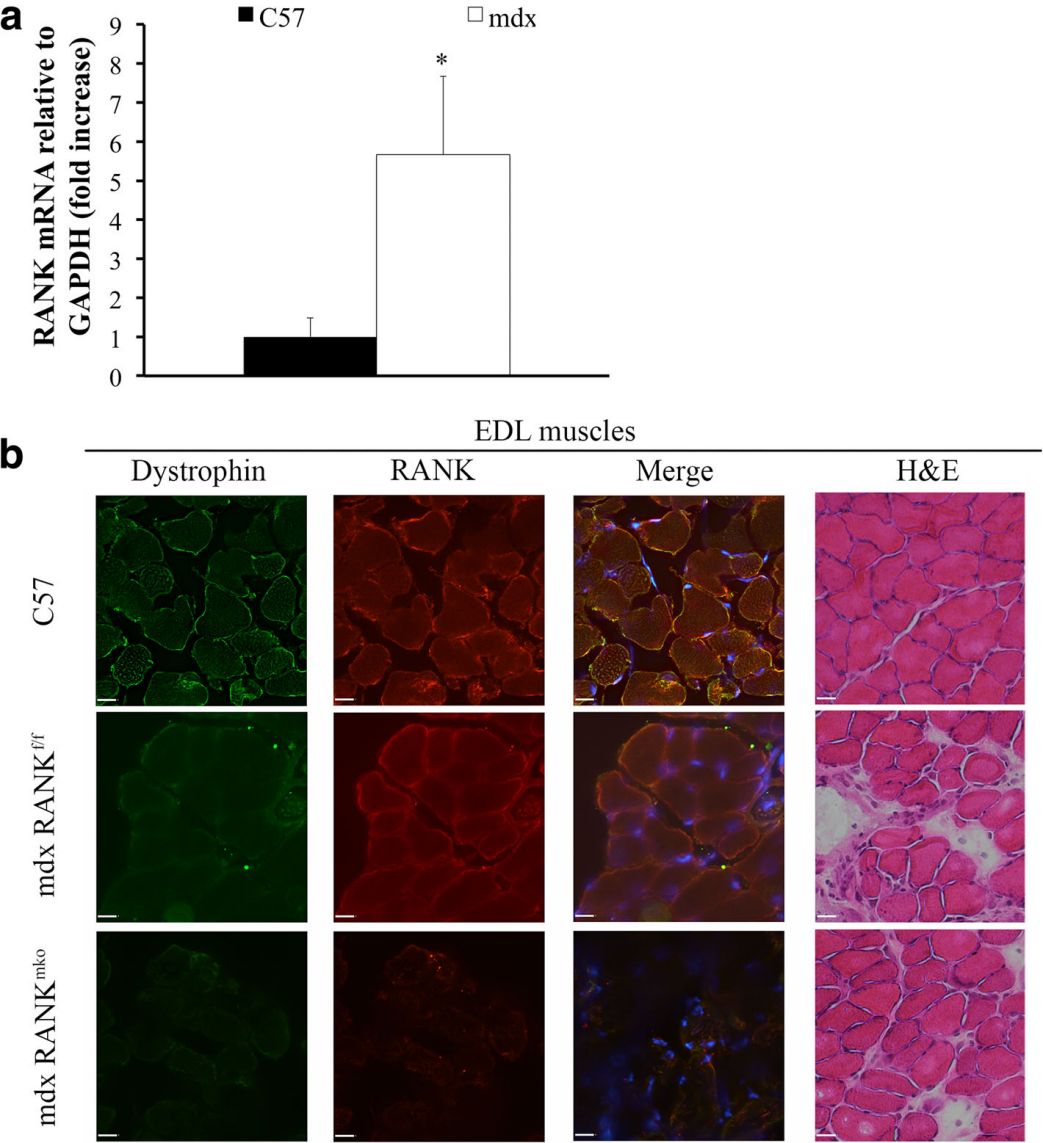
RANK expression in dystrophic skeletal muscles. **a** A quantitative real-time PCR analysis revealed that muscle RANK expression is increased by 5.5-fold in dystrophic EDL muscles relative to control. **b** Confocal images demonstrating the colocalization of dystrophin (green) and RANK (red) at the basal lamina in EDL muscles from *C57BL/6* mice. As expected, dystrophin is not expressed in EDL muscles from *mdx* mice while dystrophin and RANK proteins are absent in EDL muscles from *mdx-RANK*^*mko*^ mice. H&E staining revealed that EDL muscles from *mdx-RANK*^*mko*^ mice had a lesser number of irregular fiber sizes and accumulation of perimysial connective tissue compared with *mdx-RANK*^*f/f*^ muscles. Data are shown as mean +/− s.e.m. * Significantly different from control C57 EDL muscles, *p* < 0.05, *n* = 3, Student’s t-test. Scale bar = 100 μm

**Fig. 2:**
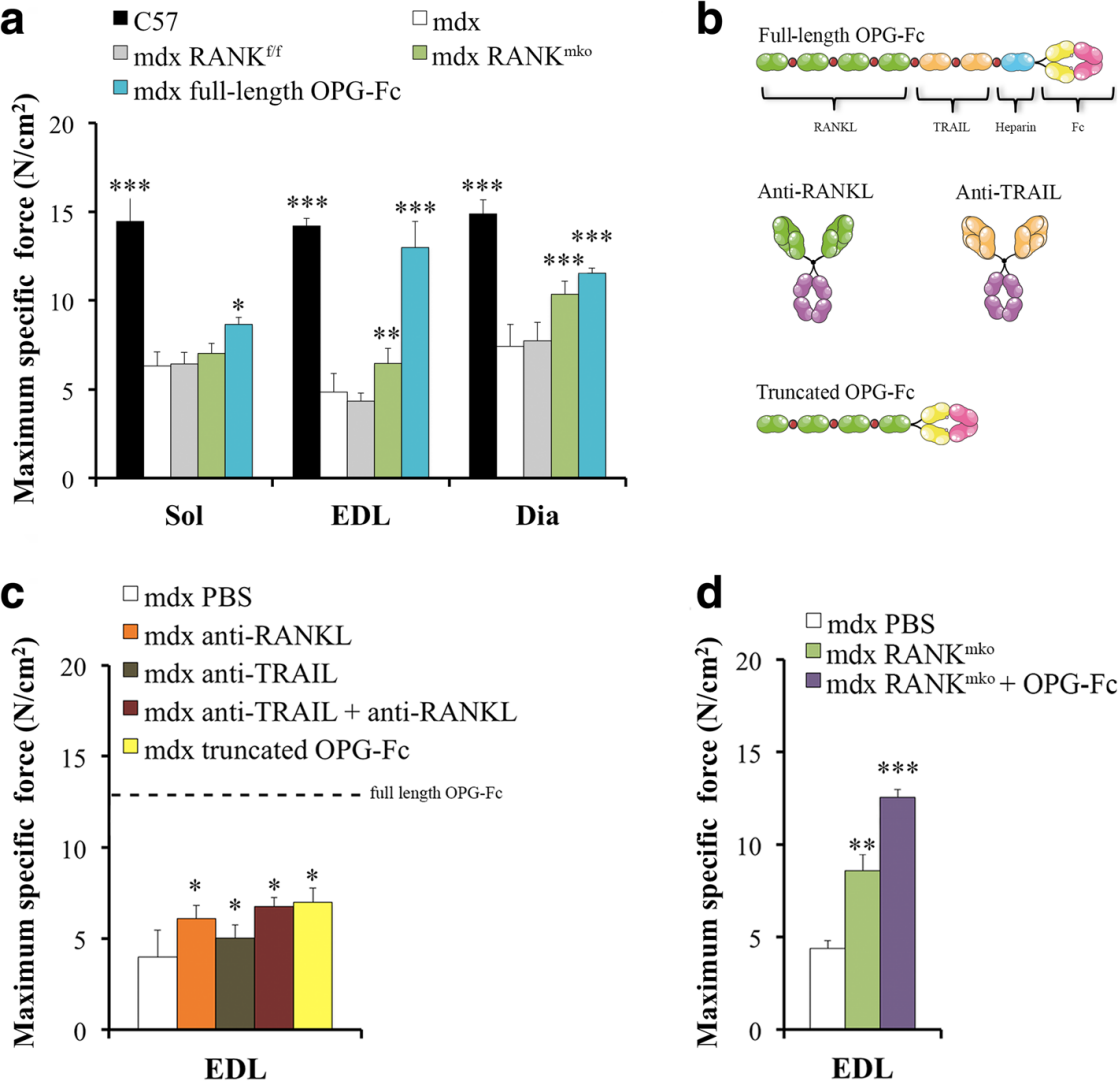
Genetic deletion of muscle RANK or selective inhibitions of RANKL or TRAIL in dystrophic mice. **a** Maximum specific force analysis of the Sol, EDL and Dia muscles were performed on 5-week-old *C57BL/6*, *mdx-RANK*^*mko*^, *mdx-RANK*^*f/f*^ *or mdx* mice treated from days 25 to 35 with vehicle (PBS) or full-length OPG-Fc [1 mg/kg/d]. Uninjured muscles from *C57BL/6* mice were used as controls. Full-length OPG-Fc [1 mg/kg/d] treatments and to a lesser extent, muscle-specific RANK deletion significantly preserved muscle force. **b** Schematic representations of full-length OPG-Fc, truncated OPG-Fc, RANKL and TRAIL antibodies. **c** Contractile properties of EDL muscles were performed on 5-week-old *mdx* mice treated from days 25 to 35 with anti-RANKL or/and anti-TRAIL antibodies or truncated OPG-Fc. The inhibition of RANKL and TRAIL for 10 days increased the force production of dystrophic EDL muscles by 45% and 17% respectively. The truncated OPG-Fc had similar effects than anti-RANKL, increasing the force production of EDL muscles by 43%, which were markedly lower than that of dystrophic EDL muscles from full-length OPG-Fc treated *mdx* mice (+ 162%). **d** To explore whether full-length OPG-Fc acts independently of RANKL/RANK, *mdx-RANK*^*mko*^ mice were treated from days 25 to 35 with full-length OPG-Fc [1 mg/kg/d], and showed additional gain in force relative to PBS-treated *mdx-RANK*^*mko*^ mice. The dotted line is the visual representation of full-length OPG-Fc data. Data are shown as mean +/− s.e.m., one way ANOVA and Tukey’s post-hoc tests; significantly different from PBS-treated *mdx* mouse; * *p* < 0.05, ** *p* < 0.01, *** *p* < 0.001

We previously showed that RANK is expressed in fully differentiated mouse myotubes, but not in proliferating C2C12 myoblasts [13]. Interestingly, RANK mRNA was 5.5 fold higher in EDL muscles from dystrophic *mdx* mice relative to *C57BL/6* mice (Fig. 1a). To explore RANK’s contribution in dystrophic skeletal muscle and compare it with OPG-Fc-mediated RANKL inhibition, we crossed dystrophic *mdx* mice with muscle-specific RANK deletion, thereafter named *mdx-RANK*^*mko*^*. Mdx-RANK*^*floxed/floxed*^ mice that do not carry the Cre recombinase, thereafter named *mdx-RANK*^*f/f*^ which appeared indistinguishable from *mdx* mice served as littermate controls. Immunohistochemistry results confirmed the presence of dystrophin in EDL muscles from *C57BL/6* mice and RANK protein on the membrane of skeletal muscle fibers from *C57BL/6* and *mdx* mice. Dystrophin and RANK proteins were not detected in skeletal muscles from *mdx-RANK*^*mko*^ mice (Fig. 1b). We tested whether muscle-specific RANK deletion improves structural integrity and muscle function. Hematoxylin/eosin staining showed that muscle RANK deletion partially preserved muscle integrity in dystrophin/RANK double-deficient mice (Fig. 1b and Additional file 1: Figure S1). The specific and absolute force production of Sol, EDL and Dia muscles from *mdx* and *mdx-RANK*^*f/f*^ mice were similar and significantly lower than *C57BL/6* mice (Fig. 2a and Additional file 1: Figure S2). Muscle-specific RANK deletion significantly increased the specific and absolute forces of dystrophic EDL muscles by 87% and 54%, respectively, when compared to the PBS-treated *mdx* mice (Fig. 2a and Additional file 1: Figure S2). Although to a lower extent than dystrophic EDL muscles, specific and absolute forces of Sol and Dia muscles were also improved in *mdx-RANK*^*mko*^ relative to *mdx-RANK*^*f/f*^ mice (Fig. 2a and Additional file 1: Figure S2). Similar gains in force were observed in 15 month-old *mdx-RANK*^*mko*^ relative to *mdx-RANK*^*f/f*^ mice (Additional file 1: Figure S3). Hematoxylin/eosin staining confirmed that muscle integrity was strongly preserved in the full-length OPG-treated *mdx* mice (Additional file 1: Figure S4). Surprisingly, OPG-Fc almost completely restored EDL muscle force to wild type levels and was significantly more effective than muscle RANK deletion during the most severe phase of muscle degeneration in *mdx* mice (Fig. 2a and Additional file 1: Figure S2). Given the high responsiveness of the EDL muscle to OPG-Fc treatment and that fast-twitch muscles are vulnerable to DMD, we chose to use this muscle for the remaining of the study.

Since full-length OPG-Fc was significantly more effective compared to muscle-specific RANK deletion in preserving muscle function during the most severe phase of degeneration in *mdx* mice, we next focused to determine the contribution of the different OPG domains to the preservation of muscle integrity (Fig. 2b). OPG is a soluble receptor for RANKL and a decoy receptor for TRAIL increasing cell survival by blocking the pro-apoptotic effects of the TRAIL/DR4–5 interaction [16, 33]. To determine the dual function of OPG and the implication of the RANKL and TRAIL domains, *mdx* mice were injected with anti-RANKL and anti-TRAIL antibodies (Fig. 2b). The inhibition of RANKL and TRAIL for 10 days increased the specific force production of dystrophic EDL muscles by 45% and 17%, respectively, which are markedly lower than full-length OPG-Fc treated *mdx* mice (162%) (Fig. 2c). Furthermore, the combination of anti-RANKL and anti-TRAIL was not superior to anti-RANKL treatment alone (Fig. 2c). To confirm the exclusive efficiency of full-length OPG-Fc, *mdx* mice were also treated with the truncated form of OPG-Fc that carries RANKL-binding domains (Fig. 2c). As expected, the truncated OPG-Fc had similar effects to anti-RANKL treatment, increasing by 48% the force production of dystrophic EDL muscles (Fig. 2c). To corroborate whether full-length OPG-Fc also acts independently of RANK/RANKL pathway, dystrophin/RANK double-deficient mice were treated with full-length OPG-Fc for 10 days. Dystrophic EDL muscles exhibited a significant gain in force (29%) relative to untreated dystrophin/RANK double-deficient mice, indicating that full-length OPG-Fc can act in part independently of the RANKL/RANK interaction (Fig. 2d). All treatments and muscle RANK deletion did not change muscle mass (Additional file 1: Table S1). Our results show that anti-RANKL, anti-TRAIL, truncated OPG-Fc treatments or RANK deletion are much less effective than full-length OPG-Fc against muscular dystrophy (Additional file 1: Figure S5).

### Full length OPG-fc, but not muscle RANK deletion, prevents eccentric contraction-induced muscle dysfunction.

**Fig. 3:**
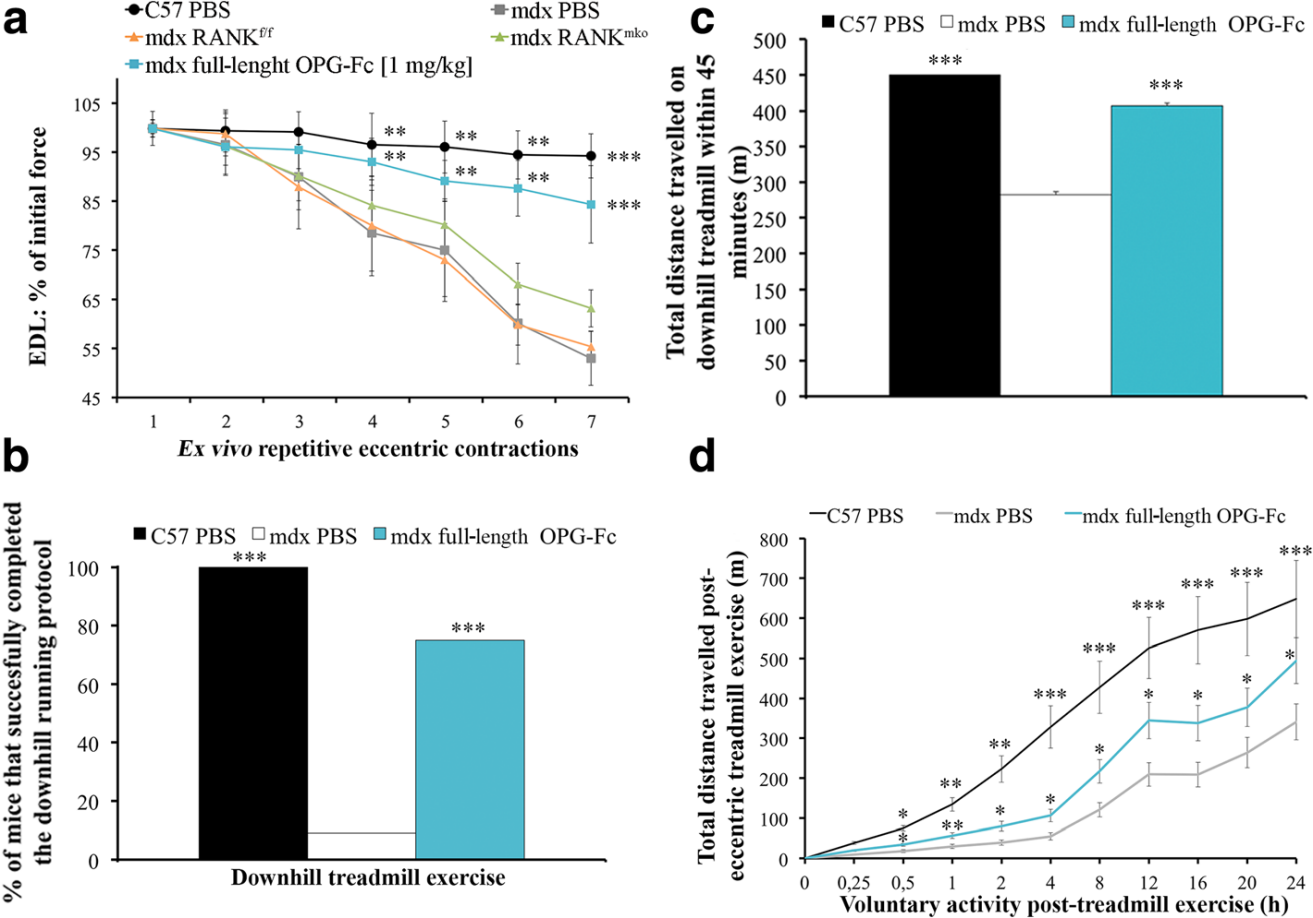
Full-length OPG-Fc, but not muscle RANK deletion, prevents eccentric contraction-induced muscle dysfunction. **a** Ex vivo eccentric contraction protocol of EDL muscles were performed on 5-week-old *C57BL/6, mdx-RANK*^*mko*^ and *mdx* mice treated from days 25 to 35 with vehicle (PBS) or full-length OPG-Fc [1 mg/kg/d]. Full-length OPG-Fc, but not muscle RANK deletion, significantly prevented the loss of force following repetitive eccentric contractions. Functional performance was assessed on 5–6-month-old *mdx* mice treated with vehicle (PBS) or full-length OPG-Fc [1 mg/kg/d] for 10 days prior downhill running. **b** Only 10% of the PBS-treated versus 75% of the full-length OPG-Fc treated *mdx* mice were able to complete the downhill running protocol at a speed of 10 m/s for 45 min. **c** The total distance travelled at 10 m/min for 45 min (450 m) for each experimental group. **d** The full-length OPG-Fc treatment rescued the voluntary cage activity post- exhausting eccentric downhill running. Data are shown as mean values +/− s.e.m.; one way ANOVA and Tukey’s post-hoc; significantly different at (**a**) each contraction or (**b**-**d**) each time point from PBS-treated *mdx* mouse; * *p* < 0.05, ** *p* < 0.01, *** *p* < 0.001

Dystrophic muscles are vulnerable to repetitive eccentric contractions [6, 17] and as opposed to muscle RANK deletion, full-length OPG-Fc positively improved EDL muscle resistance to repeated eccentric contractions (Fig. 3a), conferring a role for full-length OPG-Fc that is beyond the inhibition of RANKL/RANK interactions. To test whether the full-length OPG-Fc treatment provides a similar protection in vivo during a physiological eccentric protocol, PBS- and OPG-Fc treated adult *mdx* mice were subjected to a 45-min downhill eccentric running protocol. Ten percent of PBS-treated versus 75% of the full-length OPG-Fc treated dystrophic mice were able to complete the entire downhill running protocol (Fig. 3b). The first stop for the PBS-treated *mdx* mice occurred on average after 17 min of downhill running, while full-length OPG-Fc treated *mdx* mice stopped for the first time after 32 min (Additional file 1: Figure S6a). The PBS and full-length OPG-Fc treated mice that failed to complete the eccentric protocol did, respectively, 63% and 91% of the expected distance (Additional file 1: Figure S6b). The total distance travelled for the PBS-treated *mdx* mice was 282 m, while full-length OPG-Fc treated *mdx* mice completed 409 m (Fig. 3c). Following the strenuous eccentric protocol, mouse voluntary activity was measured by video-tracking software for 24 h. The full-length OPG-Fc treatment enhanced cage activity by roughly 50% at any given time point during the 24 h period (Fig. 3d and Additional file 1: Figure S7). Thus, our functional ex vivo and in vivo experiments provide physiological evidence that full-length OPG-Fc is very effective in protecting young and adult dystrophic mice against eccentric contraction-induced muscle dysfunction.

### Full-length OPG-fc, but not muscle-specific RANK deletion, increases SERCA activity and expression in dystrophic EDL muscles

**Fig. 4:**
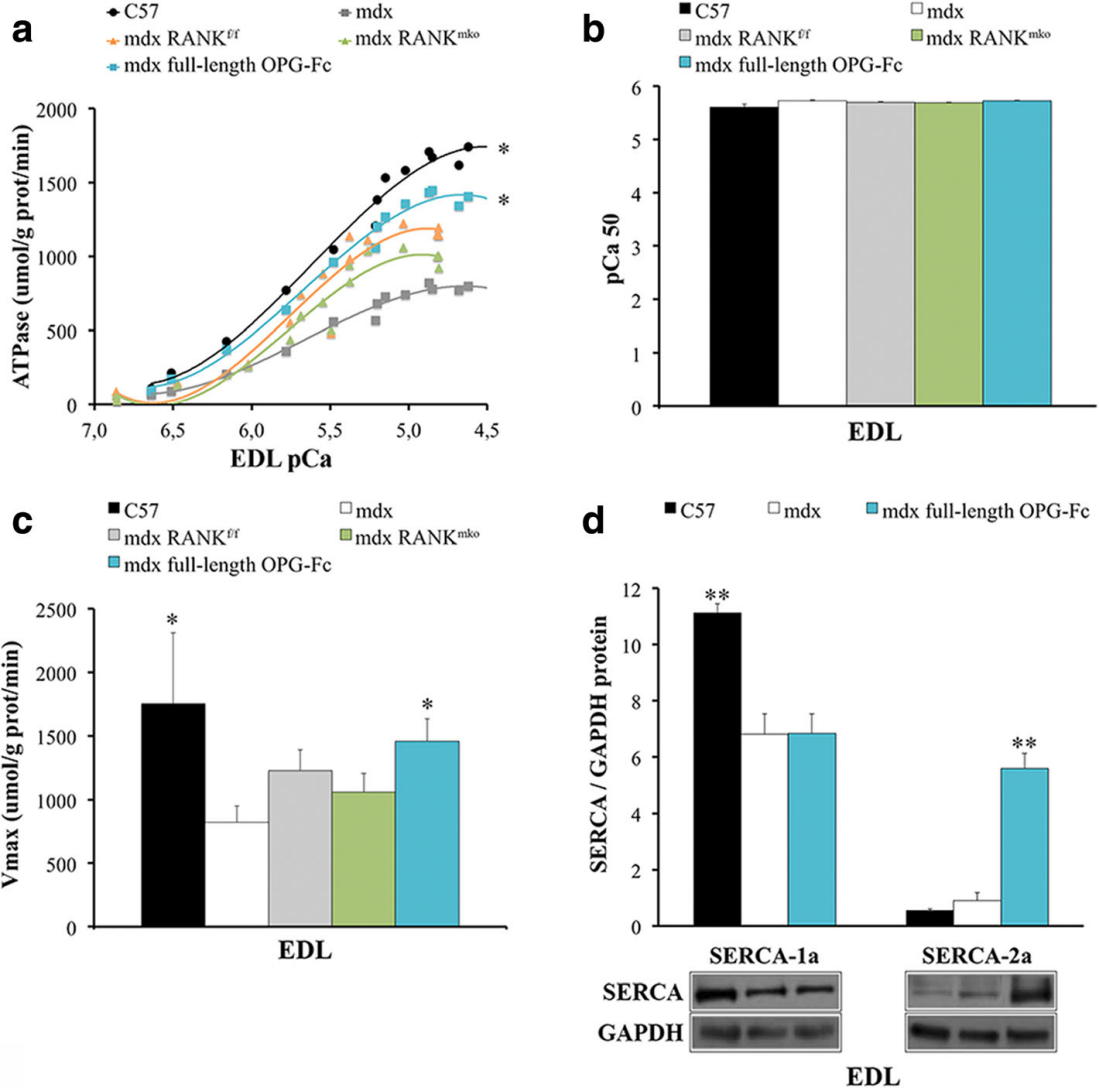
Full-length OPG-Fc treatment increased SERCA activity and SERCA-2a protein levels in fast-twitch dystrophic EDL muscles. **a** Full-length OPG-Fc [1 mg/kg/d] treatment, but not muscle RANK deletion, increased SERCA activity over different Ca^2+^ concentrations ranging from pCa 7,0–4,5 in homogenized dystrophic EDL muscles (**b**) with no change in pCa50 value. **c** Full-length OPG-Fc treatment rescued maximal ATPase activity (Vmax) in dystrophic EDL muscles**. d** Consistent with an increase in SERCA activity, full-length OPG-Fc markedly enhanced 6-fold SERCA-2a protein levels but not SERCA-1a, compared to PBS-treated *mdx* mice. Data are shown as mean values +/− s.e.m.; one way ANOVA and Tukey’s post-hoc tests; significantly different from PBS-treated *mdx* mouse; * *p* < 0.05, ** *p* < 0.01

We previously showed that muscle RANK is important in maintaining SERCA activity [13]. Since SERCA overexpression in skeletal muscles reduces susceptibility to eccentric contraction-induced muscle damage in dystrophin and sarcoglycan-null mice and given that intrinsic laryngeal muscles that overexpress SERCA are protected against muscular dystrophy [18, 29], we tested whether full-length OPG-Fc injections for 10 days might also enhance SERCA activity and SERCA-1a and SERCA-2a protein levels in fast-twitch dystrophic EDL muscles. Maximal SERCA activity is significantly depressed in dystrophic EDL muscles and full-length OPG-Fc treatment restored almost completely its activity (Fig. 4a-c). The protein levels of fast-twitch SERCA-1a were diminished in dystrophic EDL muscles and full-length OPG-Fc treatment selectively increased by 6-fold the expression of the slow-twitch SERCA-2a. (Fig. 4d). However, muscle-specific RANK deletion did not increase maximal SERCA activity in dystrophic EDL, Sol and Dia muscles (Fig. 4a-c and Additional file 1: Figure S8a-f), providing additional evidence that full-length OPG-Fc could act through alternative pathways and may potentially rescue Ca^2+^ cycling/homeostasis through SERCA-2a dependent mechanism.

## Perspective and conclusion

In the early 2000s, truncated OPG-Fc (AMGN-0007) reached clinical trial for the treatment of osteoporosis and bone metastasis [5, 7]. Truncated and full-length OPG-Fc can interact with RANKL preventing the downstream activation of NF-kB, a key controller of many genes involved in inflammation. Our observations that muscle-specific RANK deletion, anti-RANKL or truncated OPG-Fc and full-length OPG-Fc treatment protect fast-twitch fibers are of paramount importance, since these powerful fibers are the first to disappear in many forms of myopathies [28, 38]. However, we uncover a unique and superior role for full-length OPG-Fc in protecting muscle function and integrity in the *mdx* model of muscular dystrophy. Since full-length OPG-Fc rescues SERCA activity in fast-twitch dystrophic skeletal muscles, we anticipate that full-length OPG-Fc treatment would contribute to normalize SR Ca^2+^ regulation in muscular dystrophy by removing Ca^2+^ from the myoplasm and refilling the internal Ca^2+^ stores through the action of SERCA pumps. The stimulation of SERCA pumps should lead to better Ca^2+^ mobilization breaking the vicious cycle of muscle inflammation and Ca^2+^-dependent protease activation initiated by poor Ca^2+^ handling [9, 35].

**Fig. 5:**
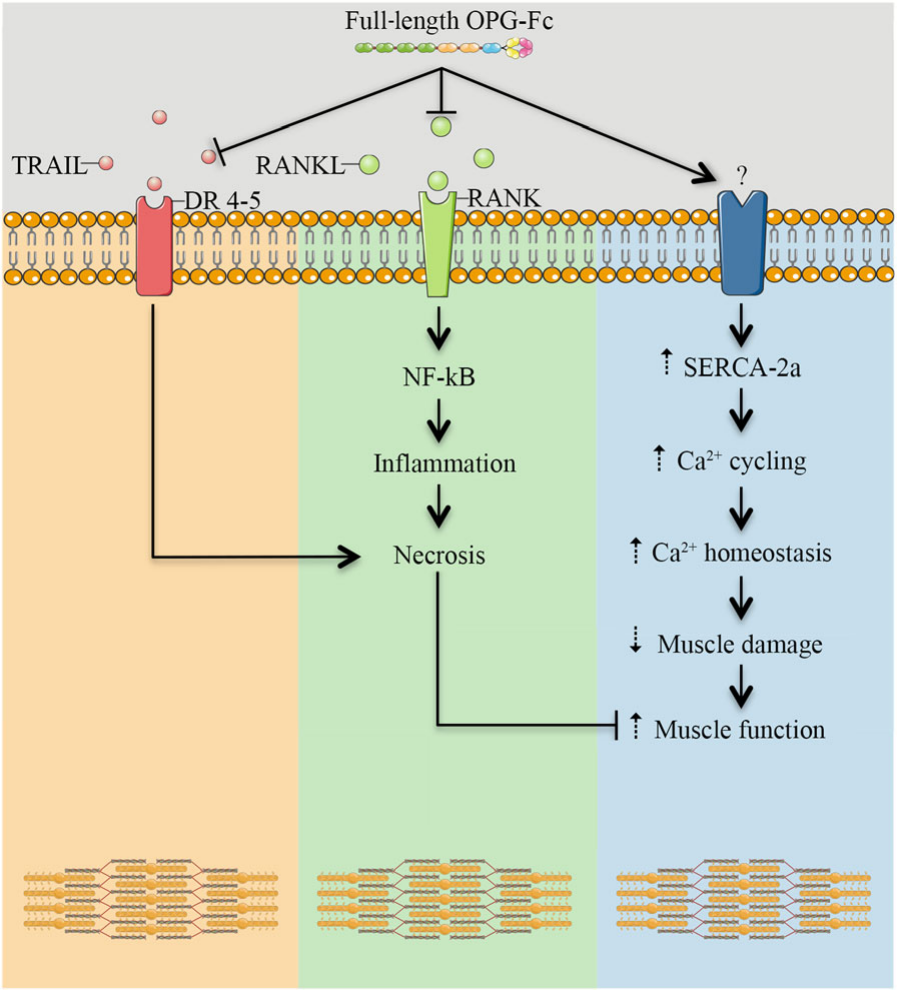
Schematic representation of the signaling pathways through which full-length OPG-Fc may exert its effects on skeletal muscle. The full-length OPG-Fc binds to RANKL and TRAIL limiting inflammation and apoptotic pathways and also binds to an unknown muscle receptor stimulating SERCA activity and Ca^2+^ entry thereby favouring Ca^2+^ cycling and homeostasis and ultimately muscle performance

Current investigations are oriented toward the various roles of full-length OPG-Fc. Bone, tumor cells, inflammatory cells and vascular cells have given some insight on how full-length OPG-Fc may work to some extent independently of RANKL inhibition in muscular dystrophy [4]. Native OPG possesses a heparin-binding domain that may interact with various glycoaminoglycans and proteoglycans and ultimately integrins [23, 24, 26, 34, 36, 40]. In addition, heparan-sulfate proteoglycans are abundant in skeletal muscles [8], increases in DMD [1] and may represent an additional target for full-length OPG-Fc [10]. On the other hand, it was recently found that integrin-linked kinase, the adaptor protein of integrins mediates force transduction in cardiomyocytes through SERCA-2a function [37]. It is thus tempting to speculate that full-length OPG-Fc-induced SERCA2a expression may also act through mutual cooperation between proteoglycans, integrins and growth factors (Fig. 5). Altogether, our results suggest that all 7 domains of OPG-Fc may contribute to prevent muscle degeneration in DMD. Since OPG is a well-known bone protector, one cannot exclude that healthier bones may also protect dystrophic muscles but this remains well beyond the scope of the present study. In conclusion, full-length OPG-Fc may be a new clinical treatment for several forms of neuromuscular and muscular diseases in which a single therapeutic approach may be foreseeable to maintain both bone and skeletal muscle functions.

## Additional file

Additional file 1: **Table 1**. All treatments and muscle-specific genetic deletion of RANK did not have an impact on muscle mass at 5 weeks of age. **Table 2**. Primers used for PCR amplification and genotyping. **Figure 1**. RANK deletion reduces EDL muscle damage in 5 week-old mdx mice. **Figure 2**. Full-length OPG-Fc treatment and RANK deletion protect dystrophic skeletal muscles. **Figure 3**. RANK deletion protects skeletal muscles in old mdx mice. **Figure 4**. Full-length OPG-Fc mitigates muscular dystrophy in fast-twitch skeletal muscles. **Figure 5**. Recovery scores of various key functional parameters of skeletal muscles evaluated ex vivo from dystrophic mice treated with full-length OPG-Fc, anti-RANKL, anti-TRAIL and/or selectively deficient in muscle RANK. **Figure 6**. Full-length OPG-Fc markedly increases functional performance during eccentric downhill running. **Figure 7**. Recovery scores of forced and voluntary physical exercise performance in full-length OPG-Fc treated dystrophic mdx mice. **Figure 8**. Muscle RANK deletion and full-length OPG-Fc treatment did not increase SERCA activity in dystrophic Sol and Dia muscles. (DOCX 3505 kb)
