# Supplementary material for: Genetic deletion of muscle RANK or selective inhibition of RANKL is not as effective as full-length OPG-fc in mitigating muscular dystrophy
Source: Acta Neuropathol Commun. 2018 Apr 24;6:31. doi: 10.1186/s40478-018-0533-1 (PMC5922009; doi:10.1186/s40478-018-0533-1)
Supplement: Supplementary file 1 — Table 1. All treatments and muscle-specific genetic deletion of RANK did not have an impact on muscle mass at 5 weeks of age. Table 2. Primers used for PCR amplification and genotyping. Figure 1. RANK deletion reduces EDL muscle damage in 5 week-old mdx mice. Figure 2. Full-length OPG-Fc treatment and RANK deletion protect dystrophic skeletal muscles. Figure 3. RANK deletion protects skeletal muscles in old mdx mice. Figure 4. Full-length OPG-Fc mitigates muscular dystrophy in fast-twitch skeletal muscles. Figure 5. Recovery scores of various key functional parameters of skeletal muscles evaluated ex vivo from dystrophic mice treated with full-length OPG-Fc, anti-RANKL, anti-TRAIL and/or selectively deficient in muscle RANK. Figure 6. Full-length OPG-Fc markedly increases functional performance during eccentric downhill running. Figure 7. Recovery scores of forced and voluntary physical exercise performance in full-length OPG-Fc treated dystrophic mdx mice. Figure 8. Muscle RANK deletion and full-length OPG-Fc treatment did not increase SERCA activity in dystrophic Sol and Dia muscles. (DOCX 3505 kb) [file 40478_2018_533_MOESM1_ESM.docx]

## Supplementary Tables, Figures and Legends

|  | **Muscle mass (mg)** | | |
| --- | --- | --- | --- |
|  | **Sol** | **EDL** | **Dia** |
| *C57* PBS | 4.53 ± 0.65 | 4.68 ± 0.34 | 14.23 ± 2.09 |
| *Mdx* PBS | 5.10 ± 0.46 | 5.01 ± 0.55 | 15.75 ± 1.44 |
| *Mdx-RANK^f/f^* | 4.89 ± 0.58 | 4.32 ± 0.64 | 14.04 ± 0.89 |
| *Mdx-RANK^mko^* | 4.78 ± 0.41 | 4.58 ± 0.39 | 14.32 ± 0.73 |
| *Mdx* full-length OPG-Fc [1 mg/kg] | 4.98 ± 0.47 | 4.52 ± 0.27 | 14.65 ± 0.59 |
| *Mdx* anti-RANKL | 4.87 ± 0.36 | 5.03 ± 0.59 | 13.96 ± 0.93 |
| *Mdx* anti-TRAIL | 4.83 ± 0.42 | 4.82 ± 0.33 | 14.55 ± 0.82 |
| *Mdx* anti-RANKL + anti-TRAIL | 4.79 ± 0.77 | 4.71 ± 0.42 | 14.62 ± 0.75 |
| *Mdx* truncated OPG-Fc | 4.66 ± 0.61 | 4.85 ± 0.38 | 14.21 ± 0.83 |
| *Mdx-RANK^mko^* + full-length OPG-Fc [1 mg/kg] | 4.84 ± 0.64 | 4.96 ± 0.51 | 14.95 ± 0.67 |

**Supplementary Table 1: All treatments and muscle-specific genetic deletion of RANK did not have an impact on muscle mass at 5 weeks of age.** Muscle mass of Sol, EDL and Dia from wild-type, *mdx-RANK^f/f^*, *mdx-RANK^mko^* and *mdx* mice treated with PBS, full-length OPG-Fc, anti-RANKL, anti-TRAIL, a combination of anti-RANKL and anti-TRAIL or truncated OPG-Fc. All data are expressed as mean ± s.e.m, one way ANOVA and Tukey’s post-hoc tests.

| **Primers** | **Sequences 5’ - 3’** |
| --- | --- |
| p87 | GGCAGAACTCGGATGCACAGATTGG |
| p88 | AGTGTGCCTGGCATGTGCAGACCTT |
| p105 | CTGGTGGTTGTTCTCCTGGTGTCAT |
| ALP 130 | CTGCCACGACCAAGTGACAGCAATG |
| ALP 131 | GCCTTCTCTACACCTGCGGTGCTAA |
| p9427 | AACTCATCAAATATGCGTGTTAGTG |
| p259E | GTCACTCAGATAGTTGAAGCCATTTAA |
| p260E | GTCACTCAGATAGTTGAAGCCATTTA |
| **Genotype condition for RANK:** | |
| p88-p105 (wild-type allele) | 256 bp fragment |
| p88-p105 (flox allele) | 390 bp fragment |
| p87-p88 (delta allele) | 566 bp fragment |
| **Genotype condition for mck-cre** | |
| ALP 130-ALP 131 (mck-cre) | 326 bp fragment |
| **Genotype condition for mdx** | |
| p9427-p259E (mdx specific allele) | 105 bp fragment |
| p9427-p260E (wild-type specific allele) | 105 bp fragment |

**Supplementary Table 2: Primers used for PCR amplification and genotyping.** RANK, cre, and dystrophin was identified by isolating genomic DNA from tail tissue and screening for the mutation or presence of the transgene by PCR. To detect delta, flox and wild- type alleles primers used were p87, p88 and p105. Conditions : 94 ^o^C 2 min, 40 cycles of (94 ^o^C 30 s, 60 ^o^C 20 s, 72 ^o^C 1 min) and 72 ^o^C 4 min. To detect the presence of the mck-cre primers used were ALP 130 and ALP 131. Conditions : 94 ^o^C 2 min, 40 cycles of (94 ^o^C 30 s, 58 ^o^C 10 s, 72 ^o^C 1 min) and 72 ^o^C 4 min. To detect mdx allele primers used were p9427 and p259E. Conditions : 94 ^o^C 3 min, 45 cycles of (94 ^o^C 30 s, 57 ^o^C 30 s, 72 ^o^C 20 s) and 72 ^o^C 10 min.

**
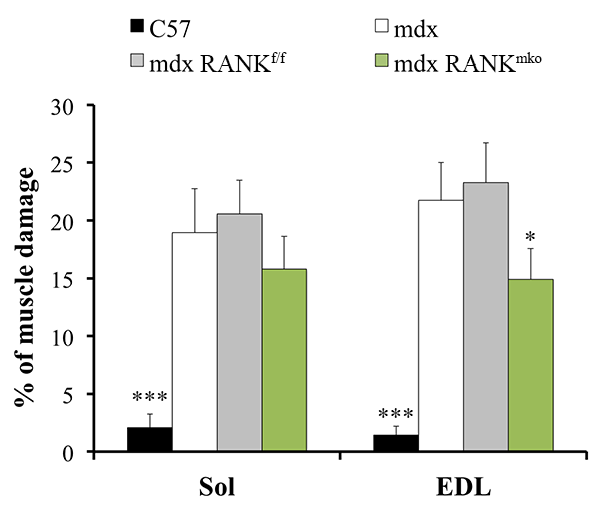
**

**Supplementary Figure 1: RANK deletion reduces EDL muscle damage in 5 week-old** ***mdx* mice.** Muscle damage quantification of Sol and EDL muscles were performed on *C57BL/6,* *mdx* mice, *mdx-RANK^f/f^* or *mdx-RANK^mko^*. Muscle RANK deletion was effective in restoring histological appearance of fast-twitch EDL, but not slow-twitch Sol muscles in 5 week-old *mdx* mice. Data are shown as mean +/- s.e.m., one way ANOVA and Tukey’s post-hoc tests; significantly different from *mdx-RANK^f/f^* mouse; * p<0.05


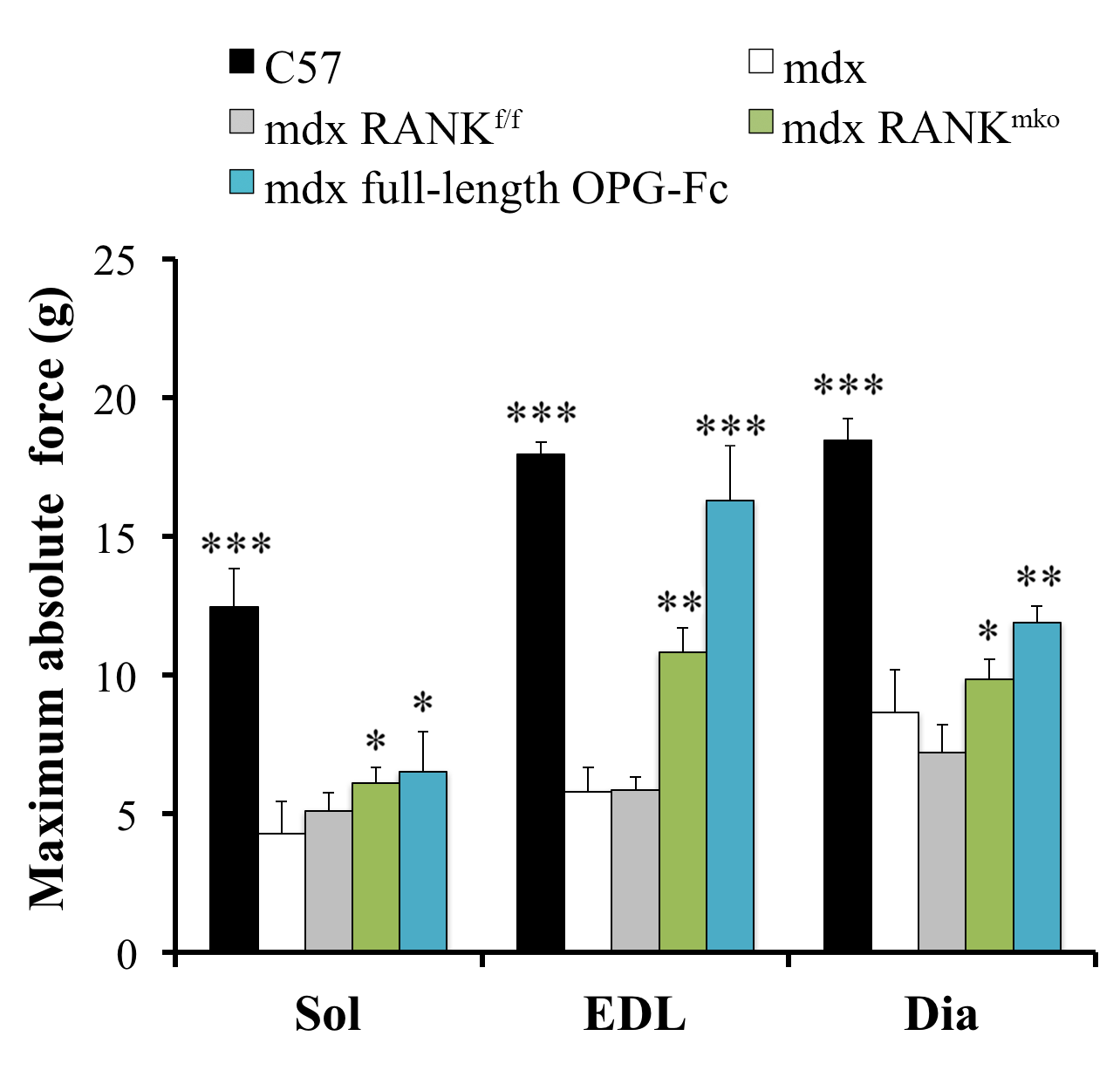


**Supplementary Figure 2: Full-length OPG-Fc treatment and RANK deletion protect dystrophic skeletal muscles.** Maximum absolute force of Sol, EDL and Dia muscles were evaluated on 5-week-old wild-type, *mdx-RANK^f/f^*, *mdx-RANK^mko^* and *mdx* mice treated from days 25 to 35 with vehicle (PBS) or full-length OPG-Fc [1 mg/kg/d]. Uninjured muscles from *C57BL/6* mice were used as controls. Although to a lower extent than full-length OPG-Fc treatment, muscle RANK deletion rescued force production, especially in fast-twitch dystrophic EDL muscles. Data are shown as mean +/- s.e.m., one way ANOVA and Tukey’s post-hoc tests; significantly different from PBS-treated *mdx* mouse; * p<0.05, ** p<0.01, *** p<0.001.


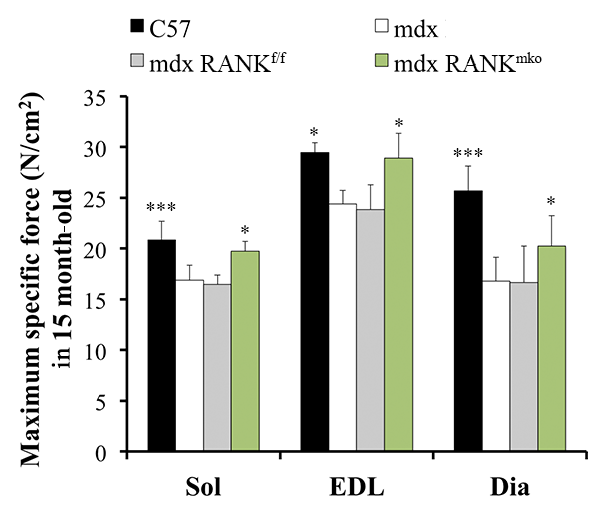


**Supplementary Figure 3: RANK deletion protects skeletal muscles in old *mdx* mice.** Maximum specific force of Sol, EDL and Dia muscles were determined on 15-month-old *C57BL/6* *mdx* mice, *mdx-RANK^f/f^* or *mdx-RANK^mko^*. Muscle RANK deletion, rescued muscle force, especially in fast-twitch EDL muscles. Data are shown as mean +/- s.e.m., one way ANOVA and Tukey’s post-hoc tests; significantly different from *mdx-RANK^f/f^* mouse; * p<0.05, *** p<0.001.

**
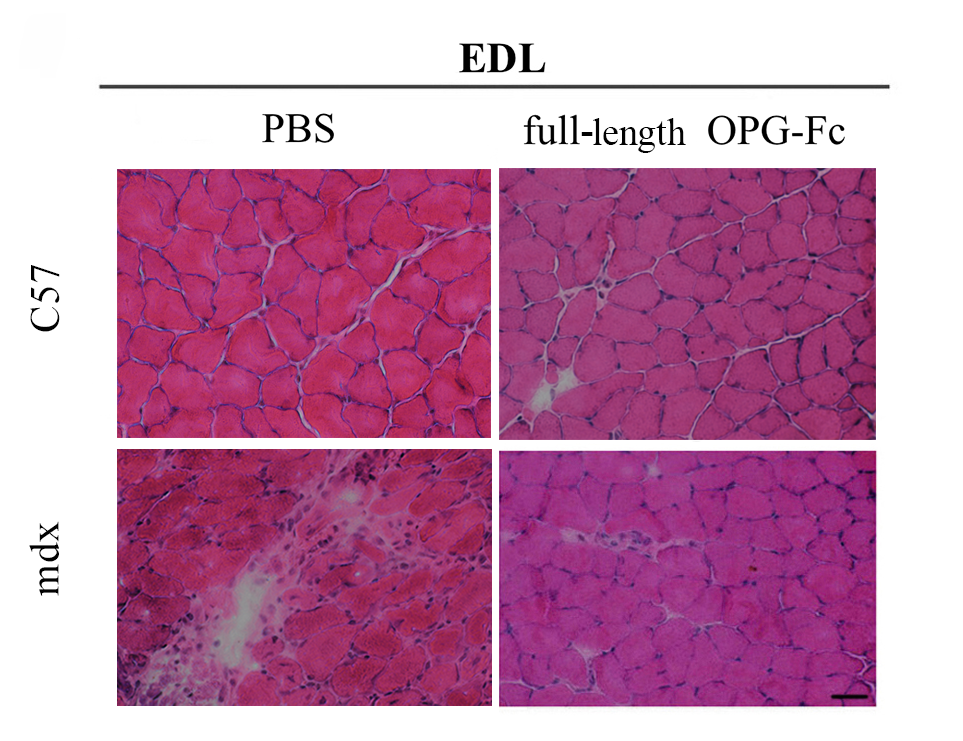
**

**Supplementary Figure 4: Full-length OPG-Fc mitigates muscular dystrophy in fast-twitch skeletal muscles.** EDL muscles from PBS-treated mdx mice showed irregular fiber sizes and focal and accumulation of perimysial connective tissue. Full-length OPG-Fc [1 mg/kg/d] treatments significantly protect the integrity and structure of dystrophic EDL muscles. Bar = 100 μm.

**
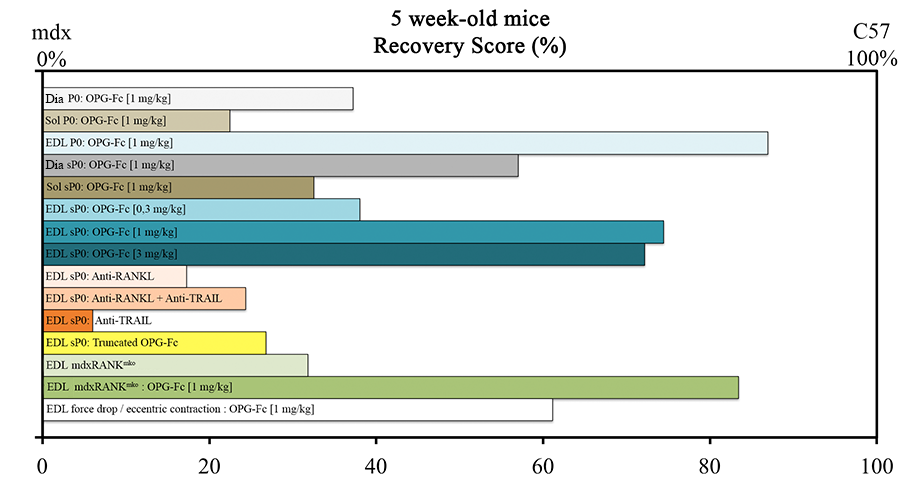
**

**Supplementary Figure 5: Recovery scores of various key functional parameters of skeletal muscles evaluated *ex vivo* from dystrophic mice treated with full-length OPG-Fc, anti-RANKL, anti-TRAIL and/or selectively deficient in muscle RANK.** The full-length OPG-Fc [1 mg/kg/d] treatment was particularly effective in restoring the function of fast-twitch EDL muscles in 5 week-old *mdx* mice. To a lesser extent, full-length OPG-Fc treatment improved function of Sol and Dia muscles. Mdx-RANK^mko^ mice treated from days 25 to 35 with full-length OPG-Fc [1 mg/kg/d] is much more effective in force production than RANK deletion alone.


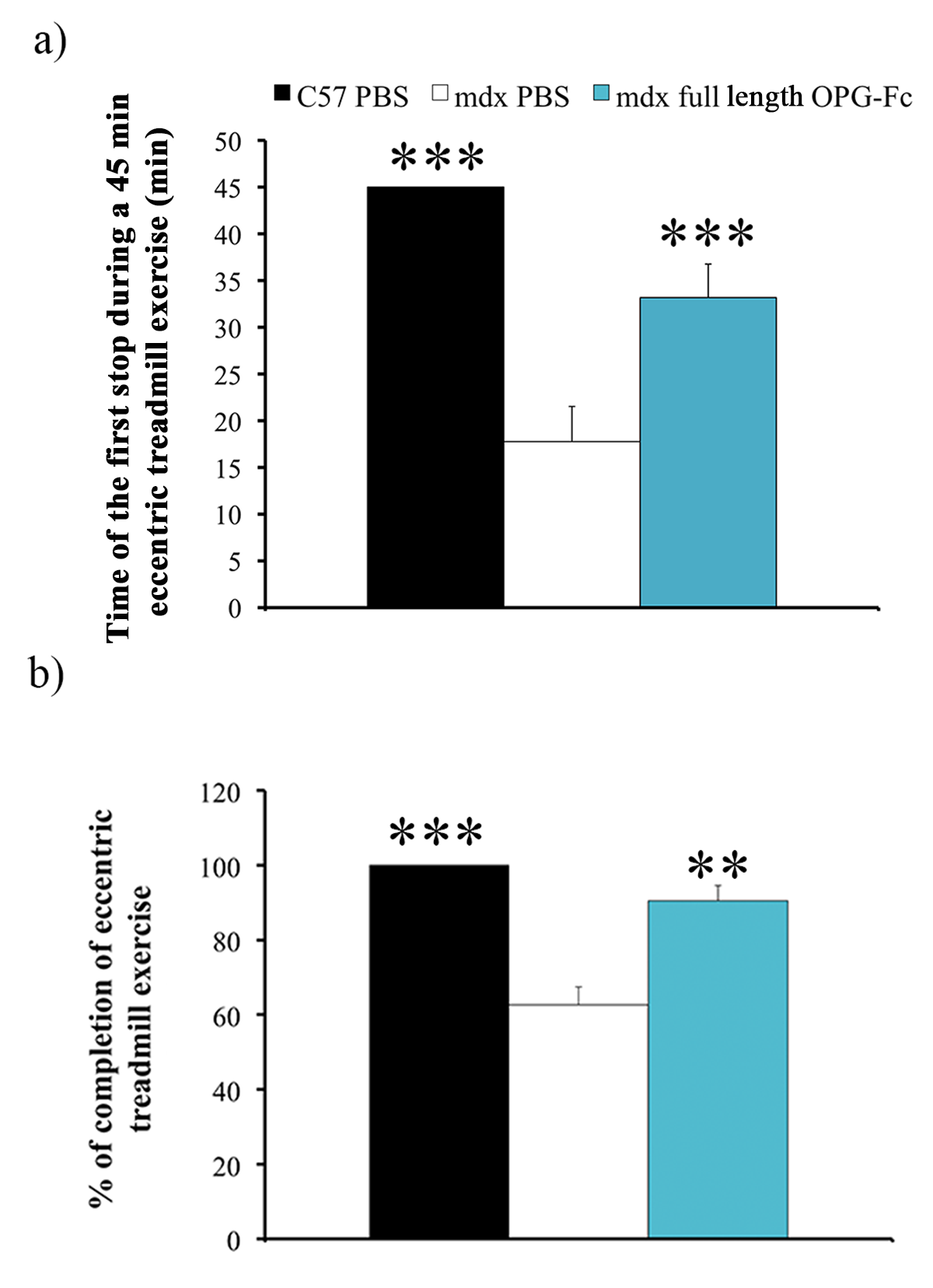


**Supplementary Figure 6**: **Full-length OPG-Fc markedly increases functional performance during eccentric downhill running.** Functional performance was assessed on 5-6-month-old *C57BL/6* and *mdx* mice treated daily with vehicle (PBS) or full-length OPG-Fc [1 mg/kg/d] for 10 d prior to downhill running (14% slope, 10 m/min for 45 min). Exhausted mice were allowed to rest for 2 min and put back on the treadmill thereafter. (**a**) The first stop for PBS-treated *mdx* mice occurred after 17 min, while the full-length OPG-Fc treated *mdx* mice stopped for the first time after 32 min of downhill running. (**b**) 91 % and 63% of the expected distance was completed respectively by the full-length OPG-Fc treated and PBS-treated *mdx* mice that failed to complete the eccentric protocol. Data are shown as mean +/- s.e.m.; one way ANOVA and Tukey’s post-hoc tests; significantly different from PBS-treated *mdx* mouse; ** p<0.01, *** p<0.001.

**
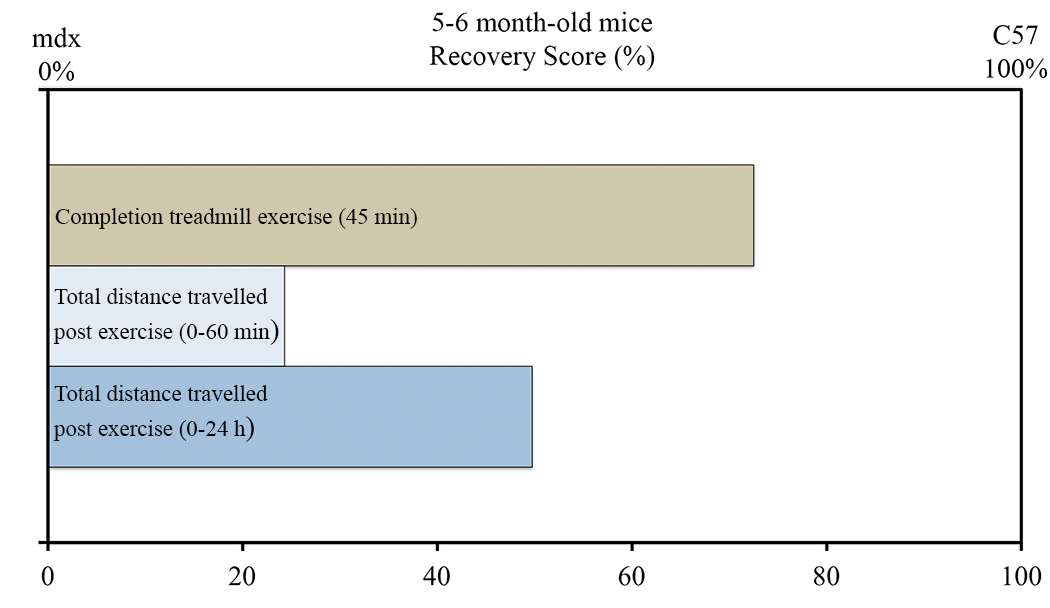
**

**Supplementary Figure 7: Recovery scores of forced and voluntary physical exercise performance in full-length OPG-Fc treated dystrophic *mdx* mice.** The full-length OPG-Fc treatment had a significant impact on running capacity during downhill treadmill exercise. Voluntary cage activity for the first 24 h post-strenuous/eccentric treadmill exercise was improved in full-length OPG-Fc treated *mdx* mice.


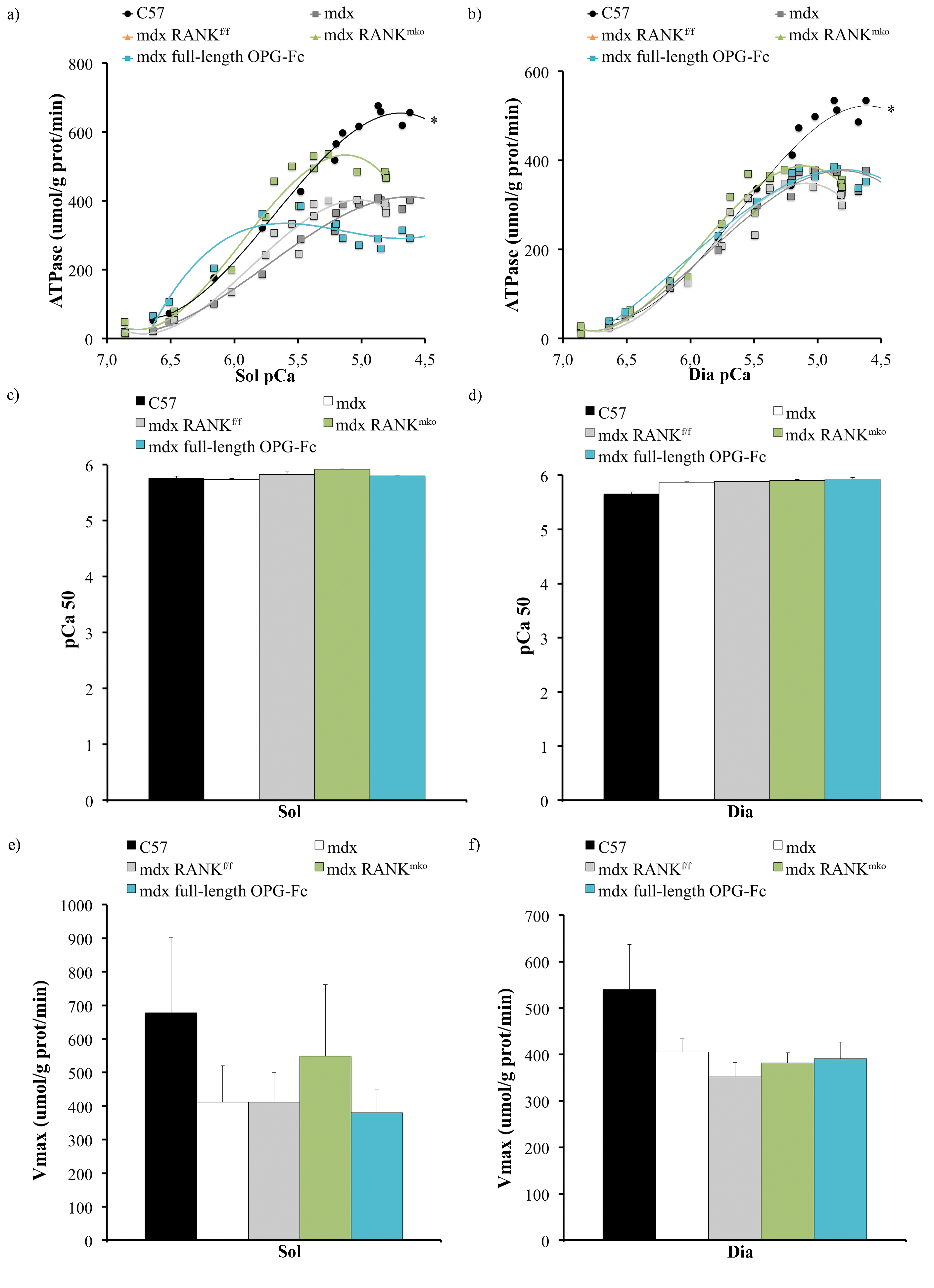


**Supplementary Figure 8**: **Muscle RANK deletion and full-length OPG-Fc treatment did not increase SERCA activity in dystrophic Sol and Dia muscles.** No differences in SERCA activity over various Ca^2+^ concentrations ranging from pCa 7,0-4,5 **(a** and **b)** with no change in pCa50 value **(c** and **d)** and maximal ATPase activity (Vmax**; e** and **f)**, in Sol and Dia muscles, respectively, of *mdx-RANK^mko^* and full-length OPG-Fc treated *mdx* mice. Data are shown as mean +/- s.e.m.; one way ANOVA and Tukey’s post-hoc tests; significantly different from PBS-treated *mdx* mouse; * p<0.05.
